# Phylogenomics of a rapid radiation: the Australian rainbow skinks

**DOI:** 10.1186/s12862-018-1130-4

**Published:** 2018-02-05

**Authors:** Jason G. Bragg, Sally Potter, Ana C. Afonso Silva, Conrad J. Hoskin, Benjamin Y. H. Bai, Craig Moritz

**Affiliations:** 1 Research School of Biology and Centre for Biodiversity Analysis, Australian National University, Canberra, Australia; 2 Herbarium of NSW, Royal Botanic Gardens & Domain Trust, Sydney, Australia; 3 cE3c - Centre for Ecology, Evolution and Environmental Changes, Faculdade de Ciências, Universidade de Lisboa, Campo Grande, 1749-016 Lisbon, Portugal; 4 College of Science & Engineering, James Cook University, Qld, Townsville, 4811 Australia; 5 Present address: Sanger Institute, Wellcome Genome Campus, Hinxton, Cambridgeshire CB10 1SA UK

**Keywords:** Skink, Lizard, Phylogenomics, Multispecies coalescent, Exon capture

## Abstract

**Background:**

The application of target capture with next-generation sequencing now enables phylogenomic analyses of rapidly radiating clades of species. But such analyses are complicated by extensive incomplete lineage sorting, demanding the use of methods that consider this process explicitly, such as the multispecies coalescent (MSC) model. However, the MSC makes strong assumptions about divergence history and population structure, and when using the full Bayesian implementation, current computational limits mean that relatively few loci and samples can be analysed for even modest sized radiations. We explore these issues through analyses of an extensive (> 1000 loci) dataset for the Australian rainbow skinks. This group consists of 3 genera and 41 described species, which likely diversified rapidly in Australia during the mid-late Miocene to occupy rainforest, woodland, and rocky habitats with corresponding diversity of morphology and breeding colouration. Previous phylogenetic analyses of this group have revealed short inter-nodes and high discordance among loci, limiting the resolution of inferred trees. A further complication is that many species have deep phylogeographic structure – this poses the question of how to sample individuals within species for analyses using the MSC.

**Results:**

Phylogenies obtained using concatenation and summary coalescent species tree approaches to the full dataset are well resolved with generally consistent topology, including for previously intractable relationships near the base of the clade. As expected, branch lengths at the tips are substantially overestimated using concatenation. Comparisons of different strategies for sampling haplotypes for full Bayesian MSC analyses (for one clade and using smaller sets of loci) revealed, unexpectedly, that combining haplotypes across divergent phylogeographic lineages yielded consistent species trees.

**Conclusions:**

This study of more than 1000 loci provides a strongly-supported estimate of the phylogeny of the Australian rainbow skinks, which will inform future research on the evolution and taxonomy of this group. Our analyses suggest that species tree estimation with the MSC can be quite robust to violation of the assumption that the individuals representing a taxon are sampled from a panmictic population.

**Electronic supplementary material:**

The online version of this article (10.1186/s12862-018-1130-4) contains supplementary material, which is available to authorized users.

## Background

Clades that have undergone recent and rapid radiations offer unique insights into the processes that drive diversification. They showcase the effects of high rates of speciation, often driven by adaptation, and help us understand these processes in ways that are applicable more generally across the tree of life [1]. A typical starting point for making inferences about these macro-evolutionary processes is a phylogeny of species [2], describing the history of population divergences leading to the extant taxa [3]. However, it can be challenging to estimate a phylogeny for rapidly radiating clades. This is because short periods between population divergences can lead to high levels of incomplete sorting of ancestral variation (incomplete lineage sorting, ILS), which manifests in phylogenetic datasets as discordance among gene trees [4].

Phylogenomic methods have the potential to assist with these inferential challenges, by generating sequence information for hundreds or thousands of loci. In principle, these large samples of independent genealogies (gene trees) allow the estimation of a phylogeny while explicitly taking account of the genealogical discordance arising from ILS. This has been formalised in an approach called the multispecies coalescent (MSC) model. The ‘full’ MSC model can be used to infer a species tree and a suite of gene trees simultaneously in a Bayesian framework, and is implemented in several major packages [5, 6]. Unfortunately, the full MSC is computationally intensive and, despite recent advances, its application to large datasets is not yet feasible [7, 8]. A number of ‘summary’ MSC approaches have been developed, where gene trees are first estimated, and these gene trees are analysed to infer the species tree [9, 10]. While these methods are often dramatically faster than the full MSC, and therefore permit analyses of larger datasets in practical computation times, they also often infer trees less accurately [7]. They also have other limitations, such as inferring topologies but not time-scaled branch lengths (e.g., ASTRAL, [11]).

A common alternative to the MSC is to simply infer a tree based on a concatenated alignment of sequences from multiple loci, which is computationally tractable, but can infer an incorrect topology where there is extensive discordance among genealogies due to ILS [12]. Concatenation analyses will also over-estimate branch lengths with a proportionally large effect towards the tips [7], because populations must coalesce at least as recently as their gene trees. This potentially has serious consequences for macro-evolutionary inference, particularly because branch lengths might be overestimated with greater bias near the tips, giving the appearance of reduced rates of diversification. Here we examine a phylogenomic dataset for a rapidly radiating clade of lizards. We analyse the data using multiple approaches, and compare the inferred topologies and branch lengths across different methods.

Australian skinks exemplify the opportunities and the challenges of studying clades that have diversified rapidly (e.g., [13–16]). Here we examine the rainbow skinks, a large clade that likely diversified rapidly during the mid-late Miocene. The three recognised genera of rainbow skinks – *Carlia*, *Lygisaurus* and *Liburnascincus* – together contain more than 60 species (http://reptile-database.reptarium.cz/; accessed 2 December, 2017). A majority of these species occur in Australia, but at least 23 recognised species (20 *Carlia* and 3 *Lygisaurus*) occur in New Guinea and Wallacea [17–20]. Resolving the number and boundaries of species in New Guinea (e.g., within the diverse *fusca* group; E. Rittmeyer, unpublished data) remains a work in progress. Therefore we focus on the Australian representatives of this clade, as for these we have a relatively robust taxonomy and extensive distributional and phylogeographic data, as well as prior phylogenetic hypotheses. The 26 recognised Australian *Carlia* species [21] are distributed across northern and eastern Australia. They inhabit leaf litter and rocky areas in diverse vegetation types from rainforest to arid areas, with the highest diversity in dry woodland habitats in tropical Australia [18]. The 11 Australian species of *Lygisaurus* occur in mesic parts of the north and east, in rainforest, woodland and rocky habitats [17]. *Liburnascincus* consists of four species that are all restricted to rocky habitats in north-eastern Australia and live a truly saxicoline lifestyle [20]. The rainbow skinks reach their highest diversity in north-east Australia, with sites that have up to 10 species occurring in close proximity.

Previous studies of the rainbow skinks have linked their habitat affinities to aspects of their morphology [22–24]; for example, *Liburnascincus* have the long-limbed morphology typical of lizards in rocky habitats [23]. The *Carlia* species have also diversified in breeding colours, with males having spectacular throat and flank breeding colours that differ among species and show some relationship to shifts to more open habitats [24]. However, efforts to understand the diversification of the rainbow skinks, including macro-evolutionary analyses of phenotypes in relation to habitat shifts, have been hindered by the difficulty of estimating a robust species tree. Studies based on mitochondrial gene sequences [25] and small numbers of nuclear loci [26] did not resolve relationships deep in the tree, including among the genera, and found discordance among loci [26]. These observations are consistent with a rapid diversification, and high levels of ILS. Resolving the phylogeny of rainbow skinks has been exacerbated by phenotypically cryptic divergence in some clades, manifesting as deeply divergent and sometimes paraphyletic phylogeographic lineages within described species [27–31], which can be strongly isolated when in secondary contact [32]. Some of these complexes have been revised taxonomically (e.g., *C. fusca* group [33]; *C. pectoralis* group [34]; *C. triacantha* group, in part [21]) but this remains to be done for other species with deep phylogeographic structure (e.g., [31]).

In this study, we explore the two major challenges in estimating the evolutionary history of rainbow skinks, both common in rapid radiations: gene tree discordance at short inter-nodes near the base of the tree, and cryptic diversity near the tips of the tree. We use exon capture sequencing [35–37] to generate a large multi-locus dataset for representatives of all named and current candidate species of the Australian rainbow skinks. We infer the relationships among species using a variety of different phylogenetic methods, including full MSC (StarBEAST2, [8]) and summary MSC (ASTRAL-II, [11]) approaches, as well as maximum likelihood estimation of concatenated alignments.

We also exploit well-characterised intra-specific lineage diversity in several focal species of *Carlia* [29, 31] to investigate the consequences of violating an assumption of MSC estimation: that the alleles representing each ‘taxon’ are drawn from a panmictic population. In many phylogenetic studies, such as ours, a modest number of samples are collected at different localities, and are aggregated to represent a ‘taxon’ when estimating the species tree. In the absence of population-level sampling, it can be difficult to determine when samples are representatives of the same panmictic population, demes that are connected by gene flow, or long-isolated lineages. To explore the consequences of systematic deviation from this assumption, we estimated species trees for a set of four closely related species of *Carlia* skinks with known, deep phylogeographic structure, drawing haplotypes for each species (*i*) from the same or (*ii*) separate individuals within an intraspecific lineage, and (*iii*) from members of divergent lineages within species.

In sum, we generate a phylogenomic dataset for the Australian rainbow skinks – a clade that has undergone a rapid radiation – and use this dataset to examine the consequences of different approaches to species tree analysis, including different methods for tree estimation, and different ways of aggregating samples into ‘taxa’ for analysis.

## Methods

### Sampling

We obtained tissue samples for all but one of the 41 currently named species of Australian *Carlia*, *Lygisaurus*, and *Liburnascincus*, (Additional file 1: Table S1), the exception being *Lygisaurus abscondita*, for which no tissue was available. This includes five named taxa, *Carlia insularis*, *Carlia isostriacantha*, *Carlia rimula*, *Carlia wundalthini* and *Lygisaurus rococo*, which have not previously appeared in a molecular phylogeny. Two of these taxa, *C. insularis* and *C. isostriacantha*, were known previously as divergent lineages of *C. johnstonei* and *C. triacantha* (respectively) [30], and described recently as separate species [21]. Where possible, we obtained at least two samples per species. For five species of *Carlia* (*C. amax, C. gracilis*, *C. munda, C. rubrigularis*, and *C. rufilatus*), we used tissue samples from multiple individuals belonging to each of two or more known phylogeographic lineages [27, 29, 31]. We also included representatives of additional candidate species, including samples of a divergent lineage similar to *Lygisaurus macfarlani* (from the Northern Territory), and two lineages of *C. rubrigularis* (northern and southern) [27]. We obtained a sample of an undescribed *C. fusca* group species from Waro, Papua New Guinea, because several Australian species have been placed in this group, which is predominantly Papuan [19]. Similarly, we obtained samples for a *Lygisaurus* lineage (cf. *curtus*) from Papua New Guinea, to provide context for our understanding of the evolutionary history of the Australian *Lygisaurus* taxa. Finally, three outgroup species were included, *Lampropholis coggeri, Lampropholis guichenoti* and *Pygmaeascincus timlowi*, each represented by two samples. In total, our dataset included 123 samples from 46 ‘taxa’, including 43 recognized species (Additional file 1: Table S1).

### Exon capture sequencing

For each sample, we performed exon capture sequencing. This method uses hybridization to a set of oligonucleotide probes to produce DNA libraries that are selectively enriched with a set of ‘target’ loci. We followed methods described by Bragg et al. [37], and the same probe set, which targets 3320 protein coding exon sequences (see Dryad repository doi:10.5061/dryad.34274 for target sequences).

We extracted DNA from each tissue sample using the salting-out method [38]. We prepared barcoded sequencing libraries for each sample using a protocol described by Meyer and Kircher [39], with modifications described by Bi et al. [36]. The barcoded DNA was pooled, along with other skink samples, in groups of 56 at a time, at equimolar concentrations, and hybridized with the probe kit (SeqCap EZ Developer Library; NimbleGen). Hybridization was performed following the manufacturer protocol, except the hybridization mix was modified slightly to contain: 1.2 μg of the pooled DNA, 5 μg of skink Cot-1 DNA (made using a method described by Trifonov et al. [40], with a sample of *Lampropholis coggeri*), and a set of 56 blocking oligos (1000 pmol). We performed Polymerase Chain Reactions (PCRs) to enrich the post-capture libraries (17 cycles).

We quantitated DNA in pooled pre- and post-capture DNA libraries (Bioanalyzer) and used qPCR to test for enrichment of target DNA, and de-enrichment of non-target DNA (following Bi et al. [36]). We then sequenced the post-capture libraries using an Illumina HiSeq (100-bp paired-end) instrument (Biomolecular Resource Facility, Australian National University). Raw sequencing reads are in the NCBI short read archive (BioProject PRJNA289283, see Additional file 1: Table S1 for BioSample numbers).

Raw sequencing reads were cleaned using a workflow that consisted of removing duplicates, merging overlapping reads, and trimming poor quality bases and adaptor sequences. This workflow is described in detail by Singhal [41], and the code used in the present study is archived in Dryad (doi.org/10.5061/dryad.v1d32). For each sample, we then assembled the clean reads using an ‘exon-specific’ approach described by Bragg et al. [37]. This workflow begins by identifying sequencing reads that are homologous to each exon (blastall 2.2.26 [42], program = blastx, expectation value = 1E-9), and performs an assembly of this small subset of reads (velvet version 1.2.08 [43] assemblies with K = 31, 41, 51, 61 and 71 were merged using cap3 version 08/06/13 [44], with parameters: -o 20 –p 99). These contigs were trimmed to the exon sequence (flanking introns removed; exonerate 2.2.0 [45]). If more than one contig was assembled for a particular target locus, the best hit to the target was identified by a reciprocal best BLAST hit criterion (blastall 2.2.26 [42]). Using these assembled target sequences for each sample as a reference, the clean sequencing reads were then mapped (bowtie2, version 2.2.4 [46]), heterozygous sites identified, and overlapping reads were used to phase heterozygous sites within target loci (Genome Analysis Toolkit, version 3.3–0-g37228af [47]). This meant that alleles at heterozygous sites could be assigned to one of two haplotype sequences (h0 and h1), though where this was not possible, the reference allele was assigned to h0 and the alternate allele to h1. Finally, the workflow produced two haplotype sequences for each locus in each sample, replacing all sites with a genotype quality score (GQ) less than 20 with an N (unknown base). The code used to call compiled software and perform other tasks in sequence assembly, mapping, calling and phasing of heterozygous sites, and creation of haplotype sequence files, is archived in Dryad (doi.org/10.5061/dryad.v1d32).

### Alignments

For each locus, we performed alignment using MACSE (v1.01b [48]). Codons were removed from alignments if they contained a site with greater than 20% missing data (trimAl v1.4.rev15 [49]). We then estimated a gene tree using RAxML (RAxML 8.2.3 [50], −m GTRGAMMA –N20), performed 100 bootstrap replicates, and used these to calculate a relative tree certainty (TC) score [51]. TC provides an index of the information content of different loci based on agreement among bootstrap replicates. Among loci, we observed a positive association between TC score and the length of the locus alignment, and with the number of parsimony informative characters (Additional file 2: Figure S3). For the purposes of analysis, we identified two sets of loci. The first set consisted of 1384 loci containing near complete data (hereafter, complete loci, or “CL”). These loci had a sequence for every sample, and each sequence was missing data for less than 35% of sites. The second set of 304 loci was a subset of the complete loci, but were also the most informative on the basis of their TC score (TC > 0.25; hereafter, complete and informative loci, or “CIL”). We did this because several recent studies have highlighted the importance of informative gene trees in species tree estimation [16, 52, 53].

### Estimating the phylogeny of the Australian rainbow skinks

In addition to exploring analytical issues, a major goal was to infer the phylogenetic tree for the Australian rainbow skinks and hence enable future comparative studies. We did this using two kinds of approaches: maximum-likelihood estimation of a tree using a concatenated alignment of multiple loci, and species tree estimation with the MSC (see Additional file 2: Figure S1 for a summary of inferential analyses).

We estimated maximum likelihood trees for concatenated alignments of the CL and CIL data using IQTREE (1.3.5 [54]). In each case, we used the best substitution model (−m TEST) based on a comparison of AICc values (calculated by IQTREE). Support for the estimated topology was inferred with rapid bootstrapping ([55], parameter: -bb 1000). For the CIL alignment, we performed a second analysis, after partitioning by locus and codon position. Here, we placed the sites at codon positions 1 and 2 of each exon in a partition, and the sites at codon position 3 in another partition, and used the hclust algorithm (described by [56], implemented in IQTREE 1.3.5 [54]) to find a ‘best-fit’ partition model via sequential merging of partitions. We did not perform a similar analysis for the CL dataset, which would have produced a very large number of possible partitions. To check that our inferred tree was supported by different implementations of maximum likelihood (identified as an issue in [53]), we also estimated trees for the CL and CIL alignments using RAxML 8.2.3 [50] (parameters -m GTRGAMMA –N20).

We applied the MSC to the rainbow skink data in two ways. First, we used a ‘summary’ coalescent method, ASTRAL-II (4.7.9 [11]), which estimates a species tree based on previously inferred gene trees for many individual loci. We began by estimating an ASTRAL species tree using the 1384 CL gene trees estimated with RAxML (8.2.3 [50], −m GTRGAMMA –N20). Node support for this tree was inferred with 100 multi-locus bootstrap replicates. However, we wanted to check that our results were robust to a range of factors that can influence phylogenetic estimation, including the implementation used for maximum likelihood estimation [53], model violation at third codon positions [57], and variation in the information content of the individual loci [16, 53]. Therefore, in addition to the RAxML gene trees (approach “R”, Additional file 2: Figure S1), we estimated gene trees for the CL loci using two approaches. First, we estimated gene trees using IQTREE (−m TEST, best model selected based on AICc; approach “Q”). Second, we generated new alignments for the CL loci with third codon positions removed, and estimated gene trees for these alignments using IQTREE (−m TEST, best model selected based on AICc; approach “Q_12_”). We estimated an ASTRAL species tree using the gene trees generated with each of the three approaches (R, Q and Q_12_). We also estimated ASTRAL species trees using gene trees (approaches R, Q and Q_12_) for the CIL subset of loci. These analyses are summarised in Additional file 2: Figure S1.

Finally, we used StarBEAST2 (version 0.6.1 [8]), an implementation of the ‘full’ MSC, to jointly infer the species tree of the rainbow skinks along with a set of gene trees, in a Bayesian Markov Chain Monte Carlo framework. This approach is very computationally intensive, such that it was only possible to run for relatively small numbers of loci. With this dataset, we had difficulty obtaining convergence for StarBEAST2 chains when using more than 32 loci. We therefore performed an ensemble of nine separate StarBEAST2 runs, using different sets of 32 loci that were selected randomly (without replacement) from the CIL alignments. Each StarBEAST2 [8] run used analytical population size integration, gene tree relaxed clocks, an HKY + Γ nucleotide substitution model (with a single κ value and a single α value shared across all the data, and four gamma rate categories), and a chain length of 2^27^ states (sampled every 2^15^ states) (for details of implementation, see example xml in dryad repository doi.org/10.5061/dryad.v1d32).

For trees inferred with MSC methods, Additional file 1: Table S1 indicates how samples were assigned to the 46 ‘taxa.’ Note that *C. triacantha* and *C. isostriacantha* were treated as a single ‘taxon’ (for MSC analyses), prior to the recent description of *C. isostriacantha* [21]. Molecular divergence between these two taxa is low, and similar to that among lineages within other species as currently recognised. As such, we do not expect this grouping to unduly distort species tree analyses (as also demonstrated below). By contrast the newly described *Carlia insularis* [21] was treated as a taxon separate from its sister, *C. johnstonei*, as there is much deeper molecular divergence between these two taxa [30].

We estimated trees for the rainbow skinks using a variety of different approaches, and we compared these trees in two ways. First, we compared topologies by calculating Robinson-Foulds distances (function ‘RF.dist’ in the R [58] package phangorn [59]). Second, we compared the branch lengths estimated by concatenation and MSC methods. We assessed this using an MSC tree whose topology was estimated using ASTRAL, with branch lengths set to the average ancestor heights from the nine StarBEAST2 posterior distributions. We then calculated the depth of each node in this tree relative to the depth of the base. We compared this to the concatenation tree that was estimated with the CL dataset, after performing a similar normalization. We forced the concatenation tree to be ultrametric (using the function ‘chronos’ in the R [58] package ape [60]), and calculated the depth of each node relative to the base. We note that concatenation trees (where each tip is a sample) contain more tips than species trees (where each tip is a ‘taxon’). To make comparison of trees meaningful, tips were dropped from concatenation trees so that each ‘taxon’ (as used in MSC analyses) was represented by one randomly-selected sample.

### Assigning alleles to taxa in the multispecies coalescent

To investigate how violation of the assumption of panmictic ‘taxa’ might affect MSC estimation of the species tree, we identified a ‘focal’ dataset, consisting of a clade of nine taxa that contained several species with well-characterized, deeply divergent lineages (see Additional file 1: Table S1). We chose samples from two diverged lineages from four species (*Carlia amax* [29]; *C. munda*, *C. rufilatus* and *C. gracilis* [31]) to use in an experiment (listed in Additional file 1: Table S1, focal clade lineage experiment). Here we used the full MSC to estimate a species tree for the clade using two alleles from each taxon, where these alleles were from: (*i*) a single individual (the two haplotypes from a single diploid; treatment “1S” or one sample), (*ii*) different individuals from the same lineage (treatment “2S1L,” or 2 samples, from 1 lineage), and (*iii*) individuals from different lineages (treatment “2S2L,” or 2 samples from 2 lineages). These treatments were chosen to reflect different kinds of violations of the assumptions that a taxon represents an undivided population. In the 1S treatment, the two haplotypes from a single individual would reflect genetic variation in a population in the unlikely event mating is random. The 2S2L treatment, where samples are drawn from different lineages, is a clear and deliberate violation of the assumption that the samples are drawn from the same population. We expect that the 2S1L treatment is a closer fit (relative to 1S and 2S1L) to the assumptions of the MSC, allowing somewhat for isolation by distance.

For each treatment, we performed nine replicate MSC analyses, each used a set of 32 loci that were randomly selected (without replacement) from among the CL alignments, and that were used for the StarBEAST2 analyses (implemented as described above for the whole clade). For each replicate, and each treatment, we chose the two alleles used in the analysis at random. That is, for treatment 1S, we chose an individual at random from among the set of candidates (indicated in Additional file 1: Table S1), and used both haplotypes from that sample in the MSC analysis. For 2S1L, we selected a lineage at random to represent each taxon, and used an allele from each of two individuals from that lineage. For the 2S2L treatment, we used an allele from one randomly chosen individual from each of two diverged lineages to represent each taxon in the MSC analysis.

## Results

We begin with a brief summary of sequencing quality and coverage statistics (Additional file 2: Figure S2). We then describe the phylogeny we inferred for the Australian rainbow skinks, with a focus on agreement and conflict among trees estimated using different approaches. We next present the results of experiments that show how the assignment of alleles to taxa can affect phylogenetic estimation under the MSC.

### Exon capture sequencing

Across the 123 samples, we assembled sequences for an average of 2606 loci, and a minimum of 2364 loci. The sequencing depth of coverage for these loci was high, with an average of 131.4 X (see Additional file 2: Figure S2). In sum, we performed high-coverage sequencing of thousands of loci, applied stringent thresholds for data completeness (resulting in the CL set, described in Methods), and performed subsequent analyses using high quality sequence data.

### Phylogenetic estimation using concatenation and species tree methods

**Fig. 1:**
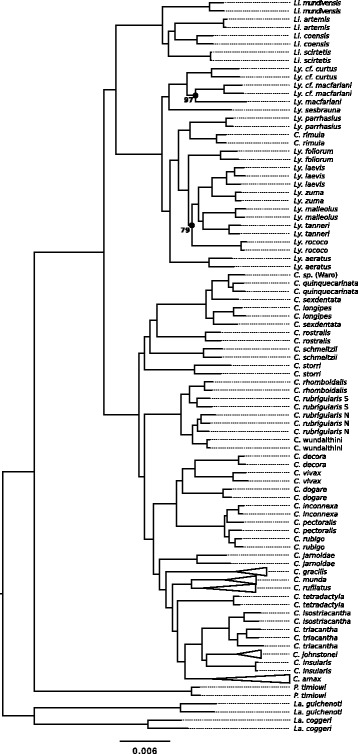
Phylogeny of the Australian rainbow skinks based on a concatenated alignment of 1384 exon sequences. This tree was estimated by maximum likelihood (implemented in IQTREE), and node labels indicate the percentage of bootstrap support (where less than 100). Clades are represented as triangles where a ‘taxon’ had more than three samples

**Fig. 2:**
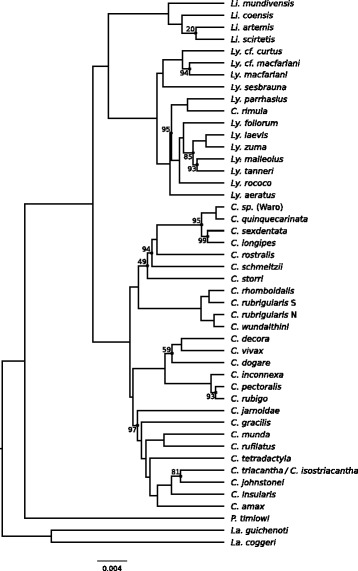
Phylogeny of the Australian rainbow skinks estimated using the multispecies coalescent (MSC). This topology was inferred using ASTRAL, based on gene trees for 304 loci (the CIL set, see text), and node labels indicate the percentage of bootstrap support (where less than 100). The branch lengths of this tree were calculated using mean ancestor heights from nine StarBEAST2 analyses, each based on a different set of 32 loci

We estimated the genetic relationships among members of the Australian rainbow skinks using a variety of approaches (see Methods, Additional file 2: Figure S1), including concatenation (Fig. 1) and multispecies coalescent (MSC) analyses (Fig. 2). The trees inferred using different approaches were often concordant, especially at nodes that were estimated with high levels of bootstrap support. Our topology also largely supports those clades that were inferred with high confidence in a previous study [26], including monophyly of the rainbow skink genera, though with interesting exceptions, which are discussed below.

**Fig. 3:**
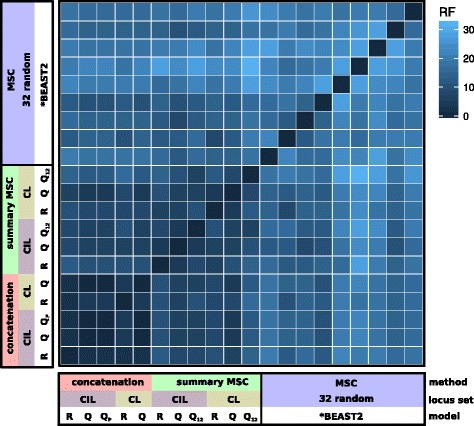
Topological discordance between phylogenies estimated using different methods. Most broadly, the different methods included concatenation, a summary MSC approach (ASTRAL) and the full MSC (StarBEAST2). We also performed analyses using different maximum likelihood implementations (RAxML, R; IQTREE, Q), partitioning (Q_P_), and analyses that excluded third codon positions (Q_12_) (see Additional file 2: Figure S1). In this matrix, the colour of each cell indicates the Robinson-Foulds distance between trees estimated using two different methods, or in the case of the StarBEAST2 trees, between one of the nine replicate analyses

The trees we inferred using concatenated alignments received high levels of bootstrap support at almost all nodes (Fig. 1). In all but three cases (*C. sexdentata*, *C. rubrigularis* and *C. pectoralis*), conspecific samples form clades, consistent with the assignments of samples to taxa used in species tree analyses (Additional file 1: Table S1). When we compare trees that were estimated using the concatenated CL versus concatenated CIL datasets, or used partitioned versus un-partitioned alignments (CIL), the topologies were highly concordant (i.e., low RF distances), except at a small number of nodes (Fig. 3).

The ASTRAL analyses also produced trees with strong bootstrap support (Fig. 2). Again, there was little difference in the topologies of the trees we inferred using the CL versus CIL datasets, or when we estimated the gene trees excluding third codon positions (Fig. 3). Overall, there was high concordance between the trees estimated using concatenated alignments, and those estimated using ASTRAL (Fig. 3).

However, the nine species trees we inferred using StarBEAST2, each based on a different set of 32 loci, often had topologies that conflicted with those estimated using ASTRAL, and with each other (Fig. 3). It is possible there is greater discordance among the StarBEAST2 analyses because each considers a much smaller sample of different genealogies (32 loci) than the concatenation and summary coalescent methods.

**Fig. 4:**
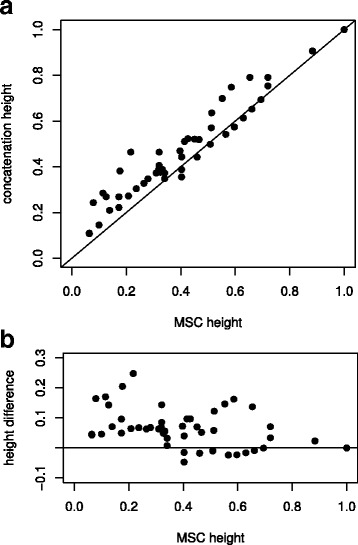
The relationship between node depths (relative to the base node) for species trees that were estimated using the multispecies coalescent (MSC) a concatenated alignment (inferred by maximum likelihood). In **a** the bivariate relationship between the inferred depths is shown, and in **b** the difference between the height inferred by concatenation and the MSC is shown, as a proportion of the depth inferred by the MSC [(concatentation – MSC) / MSC]

We also compared the branch lengths estimated by concatenation (shown in Fig. 1) and MSC (shown in Fig. 2) methods. In Fig. 4, we have plotted the relationships between the node depths estimated by the MSC (horizontal axis) and concatenation (vertical axis). We observe that many of the values lie above the line (see Fig. 4 a and b), meaning that these node depths are overestimated (relative to the base of the tree) by concatenation. This is particularly common for nodes near the tips of the tree (Fig. 4b).

### Assignment of alleles to taxa and the MSC

**Fig. 5:**
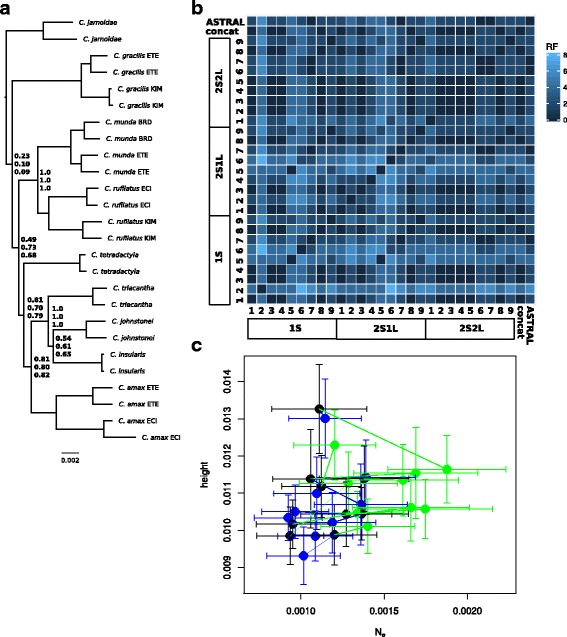
Outcomes of full MSC (StarBEAST2) analyses for a focal clade of 9 taxa of rainbow skinks, using three different ‘treatments’ for the assignment of alleles to each taxon. **a** The phylogeny, as estimated using a concatenated alignment (also shown in Fig. 1), illustrates the level of divergence between lineages in the species (*C. amax*, *C. gracilis*, *C. munda* and *C. rufilatus*) whose sampling was manipulated in the experimental treatments (1S, 2S1L, 2S2L; described in text). Support values (on the nodes of A) show the support, across the 9 replicate posterior distributions, in the species trees estimated using the 1S, 2S1L, 2S2L treatments (respectively). **b** The level of discordance (unrooted Robinson-Foulds distance) is shown among trees estimated with the 9 replicates of the tree treatments (maximum clade credibility trees), as well as the concatenation tree (A), and the topology estimated with ASTRAL (and shown in Fig. 2). **c** The tree height and *N*_*e*_ values estimated by each of the replicates, shown as the mean (± s.d.) of 100 samples from the posterior distribution of each chain. Here treatment 1S shown in blue, 2S1L in black, and 2S2L in green

In the experiment comparing sampling strategies (1S1L, 2S1L and 2S2L) there was variation in maximum clade credibility topologies among the 27 MSC analyses (Fig. 5b). However, the trees inferred across the nine replicates using the 2S2L treatment were more similar topologically to each other, and to the ASTRAL and concatenation trees, than were the trees inferred across replicates using the other two sampling approaches (Fig. 5). Additionally, the trees estimated by StarBEAST2 for the 2S2L treatment tended to have greater values for the *N*_*e*_ parameter than the other two treatments, but did not have greater tree heights (Fig. 5c).

## Discussion

### Comparing approaches of phylogenetic estimation

When we estimated the phylogeny of the Australian rainbow skinks using different approaches, the topology was affected little by several factors that have been important in other studies, such as the implementation used for maximum likelihood estimation [53], and the removal of third codon positions prior to analysis [57]. The topologies we inferred using concatenation and summary coalescent methods were also similar. While our results were largely robust to these factors, we note that this might not be true in general – for example in analyses of deeper relationships where mutational biases are more complex, or among closely related taxa where there is substantial introgression [61].

We observed greater discordance in topologies estimated with StarBEAST2, possibly because each of our StarBEAST2 analyses (necessarily) used fewer loci. This observation is consistent with the findings of Blom et al. [16], who analysed exon sequence data for a different, but also rapidly radiating, clade of Australian skinks, and found that species trees inferred using fewer than 80 loci were often discordant, but when a greater number of informative loci were used in analyses, species trees tended to converge on the same topology. With ongoing improvements in computational efficiency of StarBEAST2 [8], it should become possible to revisit full Bayesian species tree analysis for the taxa examined here.

Finally, our study corroborates previous observations that concatenation analyses overestimate the inferred depths of nodes near the tips of trees [7]. While this result was expected, it is useful to document this pattern, because it might have substantial consequences for downstream applications, such as macro-evolutionary analyses that examine the rate of divergence events through time.

### Assignment of alleles to taxa and the MSC

We found it interesting that trees generated using the 2S2L sampling strategy treatment were relatively similar to each other and to the ASTRAL and concatenation trees, given that this treatment grossly and intentionally violates the panmixia assumption of the MSC. It is possible that the co-estimation of effective population size (*N*_*e*_), as implemented in StarBEAST2, is adjusting appropriately for the extra diversity within taxa that are represented by alleles from different lineages. This possibility is supported by the observation of greater observed *N*_*e*_ values for the 2S2L treatment. In sum, these analyses show that the sampling of alleles for different taxa has consequences for trees and other parameters that are estimated with the MSC. However, the results do not add support to the concern that motivated our analysis, which was that deep intraspecific lineage diversity, and violation of relevant assumptions, might compromise phylogenetic inference. We suggest it would be worthwhile to further explore this question through a simulation study. Here datasets might be simulated for clades with known (prescribed) species trees, and where the ‘species’ contain lineages with different and contrasting divergence times. The simulated datasets could then be sampled, to test whether different sampling strategies were more likely to recover the known, true, tree.

### Phylogeny and taxonomy of the Australian rainbow skinks

In the phylogeny that we estimated for the rainbow skinks, each of the genera – *Carlia*, *Lygisaurus* and *Liburnascincus –* was monophyletic. There was an exception, in that *Carlia rimula* was placed in a clade with all members of *Lygisaurus,* as sister to *Ly. parrhasius* (Figs 1 and 2). The morphological similarity between *Carlia rimula* and *Lygisaurus parrhasius* was noted previously in a phylogenetic study [26] (see also [19]), but genetic data were not then available for *Carlia rimula*. Based on our comprehensive and well-resolved phylogeny, we reassign *C. rimula* (Ingram and Covacevich 1980) [62] to the genus *Lygisaurus*, forming the new combination *Lygisaurus rimula* comb. nov..

Among genera, *Liburnascincus* and *Lygisaurus* consistently formed a clade, to the exclusion of *Carlia* (Figs. 1 and 2). This finding is inconsistent with previous estimates of the relationship among these genera, which placed *Carlia* and *Lygisaurus* in a clade, to the exclusion of *Liburnascincus* [26, 63], albeit with low support. Our phylogenomic data now provide strong support for generic relationships.

Within each genus, the relationships we inferred among species were often concordant with previous studies, but with notable exceptions. For the genus *Carlia*, our analyses support or modify several monophyletic groups identified by Dolman and Hugall [26], which then informed a set of ‘species-groups’ that were nominated by Zug [19], and extended by Hoskin [64]. For instance, we observe a clade from north Queensland corresponding to the *fusca* species group [19], containing a New Guinea representative of the *fusca* group (*C.* Waro sp.), *C. sexdentata*, *C. longipes* and *C. quinquecarinata*. However, in contrast to previous studies, we find this group is part of a larger clade that includes *C. storri*, *C. schmeltzii* and *C. rostralis* (Fig. 1). In particular, the placement of *C. rostralis* in this clade deviates substantially from previous observations [26] and assignments [19].

The northeast Queensland rainforest-dwelling *rhomboidalis* species group [19] was supported as a clade in our analysis, consisting of *C. rhomboidalis* and *C. rubrigularis*. We also estimated a tree that places newly described *C. wundalthini* in this clade, as suggested by Hoskin [64]. We find that *C. wundalthini* is sister to the northern lineage of *C. rubrigularis*, while the southern lineage of *C. rubrigularis* is sister to *C. rhomboidalis*, confirming initial multilocus analysis by Dolman and Moritz [27]. A taxonomic revision of this group is in progress (S. Singhal et al., unpublished).

We inferred a widespread eastern Australian clade within *Carlia* containing the species *C. vivax*, *C. dogare*, *C. pectoralis*, *C. decora, C. rubigo* and *C. inconnexa.* Three of these species, *C. decora, C. rubigo* and *C. inconnexa*, were formerly included in *C. pectoralis* [34]. *Carlia rubigo* and *C. inconnexa* formed a clade with *C. pectoralis*, which supports morphological data suggesting these are three sister species [34]. The two *C. pectoralis* samples in the concatenated tree (Fig. 1) are not monophyletic; however, both ‘*C. pectoralis*’ samples come from an area of uncertainty between the known distributions of *C. pectoralis* and *C. rubigo* [34], and it is possible that sample ABTC76957 (Blackdown Tableland) is a misidentified *C. rubigo*. *Carlia decora* was sister to *C. vivax* in our phylogenies, supporting previously noted morphological similarity and species group assignment [34, 64].

Finally, for *Carlia*, we found substantial support for a clade containing the remaining nine taxa: eight of these are from the monsoonal tropics of northern Australia, but one, *C. tetradactyla*, is widespread in the southeast of the continent*.* Different methods sometimes produced conflicting estimates for relationships within this clade, particularly near its base. For instance, concatenation and summary coalescent (ASTRAL) analyses inferred strongly that *C. jarnoldae* is the sister to all other members of this clade (Figs 1 and 2), whereas StarBEAST2 analyses of this focal clade (Fig. 5a) often placed *C. gracilis* sister to the rest of the clade, or supported a clade consisting of *C. jarnoldae* and *C. gracilis*, albeit with low posterior probabilities for these clades. Other relationships were strongly supported across all analyses. We found strong and consistent support for *C. munda* and *C. rufilatus* as sister taxa, contradicting previous observations [26]. A clade containing *C. insularis*, *C. johnstonei*, and *C. triacantha* / *C. isostriacantha* was strongly supported across all analyses. Within this clade, we inferred different topologies. For instance, the concatenation analysis shown in Fig. 1 inferred that *C. johnstonei* and *C. insularis* are sister taxa (consistent with [21]), while the MSC analysis shown in Fig. 2 inferred that *C. johnstonei* and *C. triacantha* / *C. isostriacantha* are sister taxa (again with equivocal node support). Here the key point, supported by both analyses, is that the two divergence events separating these three lineages occurred in relatively quick succession.

Within *Lygisaurus*, we inferred two major clades, the smaller of which consisted of *Ly. sesbrauna*, samples allied to *Ly. macfarlani* from northern Australia, and samples allied to *Ly. curtus* from Papua New Guinea. The larger clade contained all the other sampled taxa of *Lygisaurus*, as well as ‘*Carlia rimula*’ (as noted above). This is interesting because *Lygisaurus* therefore consists of a widespread and diverse clade of north-east Australian species, occupying a variety of habitats from rainforest to arid areas and rock outcrops, and a clade that occurs in New Guinea and far north-eastern Australia. The two clades overlap in mesic forest of Cape York Peninsula.

In *Liburnascincus*, the widespread *Li. mundivensis* is most divergent, excluded from a clade containing the narrowly distributed species *Li. scirtetis*, *Li. coensis* and *Li. artemis* (Figs. 1 and 2), though the relationships within the latter clade were not well resolved in our analyses (Fig. 2). *Liburnascincus mundivensis* is found in rocky habitat over a large area of north-east Australia, whereas the other three species have highly localized distributions around boulder-fields and isolated rocky ranges on Cape York. Morphological and mtDNA similarity had previously been noted between *Li. artemis* and *Li. mundavensis* [20] but our phylogenies clearly place *Li. artemis* in a clade with the other highly localised species.

## Conclusions

This study provides a well-resolved estimate of the phylogeny of the Australian rainbow skinks. Diversification processes in this group are of great interest given their habitat and phenotypic diversity in tropical Australia, often involving many sympatric species [24]. The tree is based on data from a large number of loci, and is largely robust to different inferential approaches. It provides strong support for many previously identified clades and resolved others that have remained uncertain despite previous morphological and genetic studies. This provides the foundation for resolving the evolution and taxonomy of this group. Here we have advanced the taxonomy by formally reassigning one species (*Carlia rimula* to *Lygisaurus rimula*).

Our phylogeny also highlights the extent of deep lineage divergence within some species, often with little corresponding morphological differentiation, and this will be followed by detailed taxonomic studies. Here we explored how this intraspecific diversity might affect phylogenetic estimation, by performing an experiment where samples from diverged intraspecific lineages were deliberately placed together in ‘taxa’ for MSC analyses, violating the assumption of panmixia. We did not observe a substantial effect of this experimental treatment on the topology of inferred trees, and suggest that this might be an interesting topic for a future simulation study.

Finally, our phylogeny will provide an enhanced context for studies examining trait evolution of the rainbow skinks, their adaptation to varied habitats, and their biogeographic patterns. Additionally, we expect future studies will improve the resolution of clade memberships of *Carlia* and *Lygisaurus* from New Guinea and Wallacea, providing further insights into diversification processes across the region.

## Additional files

Additional file 1: Table S1. Information about samples used in this study. (DOCX 36 kb)

Additional file 2: Figures S1-S3. Contains figures describing our analytical workflow, and summary statistics for our sequence captures. (DOCX 264 kb)

## Abbreviations

1S: Experimental treatment – alleles from one sample
2S1L: Experimental treatment – alleles from 2 samples from 1 lineage
2S2L: Experimental treatment – alleles from 2 samples from 2 lineages
CIL: Complete and informative loci (i.e., loci with alignments containing few missing data, and that are phylogenetically informative)
CL: Complete loci (i.e., loci with alignments containing few missing data)
ILS: Incomplete lineage sorting
MSC: Multispecies coalescent
