# Supplementary material for: Phylogenomics of a rapid radiation: the Australian rainbow skinks
Source: BMC Evol Biol. 2018 Feb 5;18:15. doi: 10.1186/s12862-018-1130-4 (PMC5800007; doi:10.1186/s12862-018-1130-4)
Supplement: Supplementary file 1 — Information about samples used in this study. (DOCX 36 kb) [file 12862_2018_1130_MOESM1_ESM.docx]

Table S1. Information about samples used in this phylogenomic study of the rainbow skinks. For each sample, the genus and species is given along with a tissue number and the collection where the sample was obtained. A short read archive BioSample number is also provided. Where samples have been used in published articles previously, a citation is given. The library name of the sample (used for sequencing) is provided. The column Species also corresponds to the ‘taxon’ to which samples were assigned in MSC analyses. For samples that were used in the ‘Focal Clade’ experiment, names of corresponding lineages are provided (in the Focal Clade column).

| **Genus** | **Species** | **Tissue** | **Collection** | **BioSample (SRA)** | **Citation** | | | | **Library** | **Focal Clade** |
| --- | --- | --- | --- | --- | --- | --- | --- | --- | --- | --- |
| *Carlia* | *amax* | ABTC29892 Museum and Art Gallery of the  Northern Territory | | SAMN04420550 | Potter et al. [29] | | | | SP03_indexing25 | ECI |
| *Carlia* | *amax* | ABTC11961 | South Australian Museum | SAMN04420538 | Potter et al. [29] | | | | SP03_indexing26 | |
| *Carlia* | *amax* | ABTC72760 | South Australian Museum | SAMN04420560 | Potter et al. [29] | | | | SP03_indexing30 | |
| *Carlia* | *amax* | R36590 | Museum and Art Gallery of the Northern Territory | SAMN04420576 | Potter et al. [29] | | | | SP03_indexing36 | |
| *Carlia* | *amax* | ABTC29886 | Museum and Art Gallery of the Northern Territory | SAMN04420549 | Potter et al. [29] | | | | SP04_indexing11 | |
| *Carlia* | *amax* | ABTC29931 | Museum and Art Gallery of the Northern Territory | SAMN04420551 | Potter et al. [29] | | | | SP04_indexing17 | ECI |
| *Carlia* | *amax* | ABTC28693 | Museum and Art Gallery of the Northern Territory | SAMN04420544 | Potter et al. [29] | | | | SP04_indexing18 | |
| *Carlia* | *amax* | R114401 | Western Australian Museum | SAMN04420563 | Potter et al. [29] | | | | SP04_indexing33 | |
| *Carlia* | *amax* | R171890 | Western Australian Museum | SAMN04420569 | Potter et al. [29] | | | | SP04_indexing34 | |
| *Carlia* | *amax* | R28957 | Museum and Art Gallery of the Northern Territory | SAMN04420571 | Potter et al. [29] | | | | SP04_indexing37 | |
| *Carlia* | *amax* | A002942 | Queensland Museum | SAMN04420533 | Potter et al. [29] | | | | SP04_indexing55 | |
| *Carlia* | *amax* | ABTC72498 | South Australian Museum | SAMN05207399 Potter et al. [31] | | | | | SP11_indexing29 | ETE |
| *Carlia* | *amax* | CCM1996 | Moritz Lab ANU | SAMN05207400 Potter et al. [31] | | | | | SP11_indexing30 | ETE |
| *Carlia* | *insularis* | R117953 | Western Australian Museum | SAMN06927852 Afonso Silva et al. [30] | | | | | AS01_indexing45 | INS |
| *Carlia* | *insularis* | R117967 | Western Australian Museum | SAMN06927853 Afonso Silva et al. [30] | | | | | AS01_indexing46 | INS |
| *Carlia* | *decora* | conx5115 | Queensland Museum | SAMN05207333 | | | | | SP07_indexing4 | |
| *Carlia* | *decora* | conx5116 | Queensland Museum | SAMN05207353 | | | | | SP08_indexing4 | |
| *Carlia* | *dogare* | ABTC32199 | Queensland Museum | SAMN05207334 | | | | | SP07_indexing5 | |
| *Carlia* | *dogare* | ABTC32200 | Queensland Museum | SAMN05207354 | | | | | SP08_indexing5 | |
| *Carlia* | *gracilis* | CCM0457 | Moritz Lab ANU | SAMN05207314 Potter et al. [31] | | | | | SP05_indexing14 | |
| *Carlia* | *gracilis* | ABTC30644 | Museum and Art Gallery of the Northern Territory | SAMN04420554 | Potter et al. [29] | | | | SP05_indexing2 | ETE |
| *Carlia* | *gracilis* | CCM1135 | Moritz Lab ANU | SAMN05207316 Potter et al. [31] | | | | | SP05_indexing23 | KIM |
| *Carlia* | *gracilis* | CCM0957 | Moritz Lab ANU | SAMN05207317 Potter et al. [31] | | | | | SP05_indexing9 | KIM |
| *Carlia* | *gracilis* | ABTC11793 | South Australian Museum | SAMN05207392 Potter et al. [31] | | | | | SP11_indexing1 | ETE |
| *Carlia* | *gracilis* | ABTC28428 | Museum and Art Gallery of the Northern Territory | SAMN05207393 Potter et al. [31] | | | | | SP11_indexing10 | |
| *Carlia* | *gracilis* | CCM0958 | Moritz Lab ANU | SAMN05207406 Potter et al. [31] | | | | | SP11_indexing6 | |
| *Carlia* | *inconnexa* | J89138 | Queensland Museum | SAMN05207355 | | | | | SP08_indexing7 | |
| *Carlia* | *inconnexa* | J89132 | Queensland Museum | SAMN05207391 | | | | | SP10_indexing56 | |
| *Carlia* | *jarnoldae* | ABTC1107 | Queensland Museum | SAMN05207336 | | | | | SP07_indexing8 | JAR |
| *Carlia* | *jarnoldae* | ABTC77191 | South Australian Museum | SAMN05207356 | | | | | SP08_indexing8 | JAR |
| *Carlia* | *johnstonei* | R171237 | Western Australian Museum | SAMN06927836 Afonso Silva et al. [30] | | | | | AS01_indexing29 | |
| *Carlia* | *johnstonei* | CCM0934 | Moritz Lab ANU | SAMN06927837 Afonso Silva et al. [30] | | | | | AS01_indexing30 | JOH |
| *Carlia* | *johnstonei* | R117726 | Western Australian Museum | SAMN06927838 Afonso Silva et al. [30] | | | | | AS01_indexing31 | JOH |
| *Carlia* | *johnstonei* | MCZA28608 | Western Australian Museum | SAMN06927854 Afonso Silva et al. [30] | | | | | AS01_indexing47 | |
| *Carlia* | *longipes* | ABTC11002 | Australian Museum | SAMN05207337 | | | | | SP07_indexing9 | |
| *Carlia* | *longipes* | ABTC51118 South Australian Museum | | SAMN05207357 | | | | | SP08_indexing9 | |
| *Carlia* | *munda* | R131750 | Western Australian Museum | SAMN05207312 Potter et al. [31] | | | | | SP04_indexing43 | |
| *Carlia* | *munda* | ABTC10845 | Australian Museum | SAMN05207318 Potter et al. [31] | | | | | SP07_indexing10 | BRD |
| *Carlia* | *munda* | ABTC10846 | Australian Museum | SAMN05207338 Potter et al. [31] | | | | | SP08_indexing10 | BRD |
| *Carlia* | *munda* | ABTC102927 | Queensland Museum | SAMN05207401 Potter et al. [31] | | | | | SP11_indexing33 | |
| *Carlia* | *munda* | ABTC72508 | Museum and Art Gallery of the Northern Territory | SAMN05207402 Potter et al. [31] | | | | | SP11_indexing44 | |
| *Carlia* | *munda* | ABTC28277 | Museum and Art Gallery of the Northern Territory | SAMN05207403 Potter et al. [31] | | | | | SP11_indexing45 | |
| *Carlia* | *munda* | ABTC10977 | Australian Museum | SAMN05207404 Potter et al. [31] | | | | | SP11_indexing46 | ETE |
| *Carlia* | *munda* | ABTC28960 | Museum and Art Gallery of the Northern Territory | SAMN05207405 Potter et al. [31] | | | | | SP11_indexing47 | ETE |
| *Carlia* | *pectoralis* | ABTC76882 | South Australian Museum | SAMN05207322 | | | | | SP07_indexing14 | |
| *Carlia* | *pectoralis* | ABTC76957 | South Australian Museum | SAMN05207342 | | | | | SP08_indexing14 | |
| *Carlia* | *quinquecarinata* | ABTC102373 | Queensland Museum | SAMN05207323 | | | | | SP07_indexing15 | |
| *Carlia* | *quinquecarinata* | ABTC102374 | Queensland Museum | SAMN05207343 | | | | | SP08_indexing15 | |
| *Carlia* | *rhomboidalis* | ABTC80487 South Australian Museum | | SAMN05207387 | | | | | SP10_indexing20 | |
| *Carlia* | *rhomboidalis* | ABTC14188 | South Australian Museum | SAMN05207388 | | | | | SP10_indexing28 | |
| *Carlia* | *rostralis* | A006771 | Queensland Museum | SAMN05207372 | | | | | SP09_indexing26 | |
| *Carlia* | *rostralis* | A006581 | Queensland Museum | SAMN05207373 | | | | | SP09_indexing27 | |
| *Carlia* | *rubigo* | J89141 | Queensland Museum | SAMN05207325 | | | | | SP07_indexing17 | |
| *Carlia* | *rubigo* | J89142 | Queensland Museum | SAMN05207345 | | | | | SP08_indexing17 | |
| *Carlia* | *rubrigularis* North | SS33 | Moritz lab ANU | SAMN03787456 | | | Potter et al. [29] | | SP03_indexing6 | |
| *Carlia* | *rubrigularis* North | ABTC11016 | Australian Museum | SAMN05207324 | | | | | SP07_indexing16 | |
| *Carlia* | *rubrigularis* North | ABTC11021 | Australian Museum | SAMN05207344 | | | | | SP08_indexing16 | |
| *Carlia* | *rubrigularis* South | SS46 | Moritz lab ANU | SAMN03787434 | | Bragg et al. [37] | | | SP02A_indexing7 | |
| *Carlia* | *rubrigularis* South | ABTC77086 South Australian Museum | | SAMN05207386 | | | | | SP10_indexing18 | |
| *Carlia* | *rufilatus* | CMWA35 | Moritz Lab ANU | SAMN05207313 Potter et al. [31] | | | | | SP04_indexing9 | KIM |
| *Carlia* | *rufilatus* | R27658 | Museum and Art Gallery of the Northern Territory | SAMN05207315 Potter et al. [31] | | | | | SP05_indexing16 | |
| *Carlia* | *rufilatus* | ABTC29321 Museum and Art Gallery of the  Northern Territory | | SAMN05207394 Potter et al. [31] | | | | | SP11_indexing16 | ECI |
| *Carlia* | *rufilatus* | ABTC29295 Museum and Art Gallery of the  Northern Territory | | SAMN05207395 Potter et al. [31] | | | | | SP11_indexing17 | ECI |
| *Carlia* | *rufilatus* | CCM0677 | Moritz Lab ANU | SAMN05207396 Potter et al. [31] | | | | | SP11_indexing22 | |
| *Carlia* | *rufilatus* | ABTC29941 Museum and Art Gallery of the  Northern Territory | | SAMN05207397 Potter et al. [31] | | | | | SP11_indexing24 | |
| *Carlia* | *rufilatus* | ABTC28405 | Australian Museum | SAMN05207398 Potter et al. [31] | | | | | SP11_indexing28 | KIM |
| *Carlia* | *schmeltzii* | ABTC11024 | Australian Museum | SAMN05207326 | | | | | SP07_indexing18 | |
| *Carlia* | *schmeltzii* | ABTC77118 | South Australian Museum | SAMN05207346 | | | | | SP08_indexing18 | |
| *Carlia* | *sexdentata* | ABTC10982 Australian Museum | | SAMN05207327 | | | | | SP07_indexing19 | |
| *Carlia* | *sexdentata* | ABTC10985 | Australian Museum | SAMN05207347 | | | | | SP08_indexing19 | |
| *Carlia* | *sp.* (Waro) | ABTC44734 | Australian Museum | SAMN05207335 | | | | | SP07_indexing6 | |
| *Carlia* | *storri* | A010492 | Queensland Museum | SAMN05207380 | | | | | SP09_indexing40 | |
| *Carlia* | *storri* | ABTC53601 | South Australian Museum | SAMN05207389 | | | | | SP10_indexing29 | |
| *Carlia* | *tetradactyla* | ABTC11042 | Australian Museum | SAMN05207328 | | | | | SP07_indexing20 | TET |
| *Carlia* | *tetradactyla* | ABTC17099 | South Australian Museum | SAMN05207348 | | | | | SP08_indexing20 | TET |
| *Carlia* | *isostriacantha** | R168590 | Western Australian Museum | SAMN06927823 Afonso Silva et al. [21] | | | | | AS01_indexing16 | |
| *Carlia* | *triacantha* | R139010 | Western Australian Museum | SAMN06927841 Afonso Silva et al. [21] | | | | | AS01_indexing34 | TRI |
| *Carlia* | *triacantha* | ABTC29091 | South Australian Museum | SAMN06927846 Afonso Silva et al. [21] | | | | | AS01_indexing39 | |
| *Carlia* | *triacantha* | CCM1859 | Northern Territory Museum | SAMN06927848 Afonso Silva et al. [21] | | | | | AS01_indexing41 | TRI |
| *Carlia* | *isostriacantha** | ABTC29851 | South Australian Museum | SAMN06927812 Afonso Silva et al. [21] | | | | | AS01_indexing5 | |
| *Carlia* | *vivax* | A006791 | Queensland Museum | SAMN05207376 | | | | | SP09_indexing3 | |
| *Carlia* | *vivax* | A007267 | Queensland Museum | SAMN05207379 | | | | | SP09_indexing4 | |
| *Carlia* | *wundalthini* | conx5328 | Hoskin collection | SAMN05207374 | | | | | SP09_indexing28 | |
| *Carlia* | *wundalthini* | conx5330 | Hoskin collection | SAMN05207382 | | | | | SP09_indexing56 | |
| *Lampropholis* | *coggeri* | SS60 | Moritz lab ANU | SAMN03787435 | | Bragg et al. [37] | | | SP02A_indexing4 | |
| *Lampropholis* | *coggeri* | SEW8451 | Moritz lab ANU | SAMN03787429 | | Bragg et al. [37] | | | SP02A_indexing5 | |
| *Lampropholis* | *guichenoti* | ABTC12335 | South Australian Museum | SAMN05207329 | | | | | SP07_indexing28 | |
| *Lampropholis* | *guichenoti* | ABTC79668 | South Australian Museum | SAMN05207349 | | | | | SP08_indexing28 | |
| *Liburnascincus* | *artemis* | conx5371 | Hoskin collection | SAMN05207375 | | | | | SP09_indexing29 | |
| *Liburnascincus* | *artemis* | conx5373 | Hoskin collection | SAMN05207377 | | | | | SP09_indexing30 | |
| *Liburnascincus* | *coensis* | A004566 | Queensland Museum | SAMN05207365 | | | | | SP09_indexing17 | |
| *Liburnascincus* | *coensis* | A002088 | Queensland Museum | SAMN05207366 | | | | | SP09_indexing18 | |
| *Liburnascincus* | *mundivensis* | ABTC10839 | Australian Museum | SAMN05207319 | | | | | SP07_indexing11 | |
| *Liburnascincus* | *mundivensis* | ABTC77119 | South Australian Museum | SAMN05207339 | | | | | SP08_indexing11 | |
| *Liburnascincus* | *scirtetis* | A002000 | Queensland Museum | SAMN05207367 | | | | | SP09_indexing19 | |
| *Liburnascincus* | *scirtetis* | A004721 | Queensland Museum | SAMN05207369 | | | | | SP09_indexing20 | |
| *Lygisaurus* | *aeratus* | ABTC10855 | Australian Museum | SAMN05207381 | | | | | SP09_indexing5 | |
| *Lygisaurus* | *aeratus* | A007261 | Queensland Museum | SAMN05207383 | | | | | SP09_indexing6 | |
| *Lygisaurus* | *cf. curtus* | ABTC44460 | Australian Museum | SAMN05207320 | | | | | SP07_indexing12 | |
| *Lygisaurus* | *cf. curtus* | ABTC46164 | Australian Museum | SAMN05207340 | | | | | SP08_indexing12 | |
| *Lygisaurus* | *cf. macfarlani* | ABTC30000 | Museum and Art Gallery of the Northern Territory | SAMN05207331 | | | | | SP07_indexing30 | |
| *Lygisaurus* | *cf. macfarlani* | ABTC29123 | Museum and Art Gallery of the Northern Territory | SAMN05207351 | | | | | SP08_indexing30 | |
| *Lygisaurus* | *macfarlani* | conx5614 | Hoskin collection | SAMN05207378 | | | | | SP09_indexing39 | |
| *Lygisaurus* | *foliorum* | ABTC10971 | Australian Museum | SAMN05207330 | | | | | SP07_indexing29 | |
| *Lygisaurus* | *foliorum* | ABTC72910 | South Australian Museum | SAMN05207350 | | | | | SP08_indexing29 | |
| *Lygisaurus* | *laevis* | SEW8024 | Moritz lab ANU | SAMN03787451 | | | | Bragg et al. 2016 [37] | SP03_indexing17 | |
| *Lygisaurus* | *laevis* | A001848 | Queensland Museum | SAMN05207359 | | | | | SP09_indexing10 | |
| *Lygisaurus* | *laevis* | A000355 | Queensland Museum | SAMN05207360 | | | | | SP09_indexing11 | |
| *Lygisaurus* | *malleolus* | A006602 | Queensland Museum | SAMN05207361 | | | | | SP09_indexing12 | |
| *Lygisaurus* | *malleolus* | A006770 | Queensland Museum | SAMN05207362 | | | | | SP09_indexing13 | |
| *Lygisaurus* | *parrhasius* | ABTC31977 | Queensland Museum | SAMN05207321 | | | | | SP07_indexing13 | |
| *Lygisaurus* | *parrhasius* | ABTC31978 | Queensland Museum | SAMN05207341 | | | | | SP08_indexing13 | |
| *Carlia* | *rimula* | A004565 | Queensland Museum | SAMN05207358 | | | | | SP09_indexing1 | |
| *Carlia* | *rimula* | A004595 | Queensland Museum | SAMN05207368 | | | | | SP09_indexing2 | |
| *Lygisaurus* | *rococo* | LR7 | A. Pintor collection | SAMN05207332 | | | | | SP07_indexing31 | |
| *Lygisaurus* | *rococo* | LR11 | A. Pintor collection | SAMN05207352 | | | | | SP08_indexing31 | |
| *Lygisaurus* | *sesbrauna* | A004711 | Queensland Museum | SAMN05207363 | | | | | SP09_indexing15 | |
| *Lygisaurus* | *tanneri* | A004762 | Queensland Museum | SAMN05207364 | | | | | SP09_indexing16 | |
| *Lygisaurus* | *tanneri* | ABTC32197 | Queensland Museum | SAMN05207390 | | | | | SP10_indexing30 | |
| *Lygisaurus* | *zuma* | A000129 | Queensland Museum | SAMN05207384 | | | | | SP09_indexing8 | |
| *Lygisaurus* | *zuma* | A007790 | Queensland Museum | SAMN05207385 | | | | | SP09_indexing9 | |
| *Pygmaeascincus* | *timlowi* | A001585 | Queensland Museum | SAMN05207370 | | | | | SP09_indexing21 | |
| *Pygmaeascincus* | *timlowi* | A007378 | Queensland Museum | SAMN05207371 | | | | | SP09_indexing22 | |

* In StarBEAST2 analyses of the rainbow skink clade, these samples were included in the ‘taxon’ *Carlia triacantha*, prior to the description of *Carlia isostriacantha*.
