# Supplementary material for: Phylogenomics of a rapid radiation: the Australian rainbow skinks
Source: BMC Evol Biol. 2018 Feb 5;18:15. doi: 10.1186/s12862-018-1130-4 (PMC5800007; doi:10.1186/s12862-018-1130-4)
Supplement: Supplementary file 2 — Contains figures describing our analytical workflow, and summary statistics for our sequence captures. (DOCX 264 kb) [file 12862_2018_1130_MOESM2_ESM.docx]

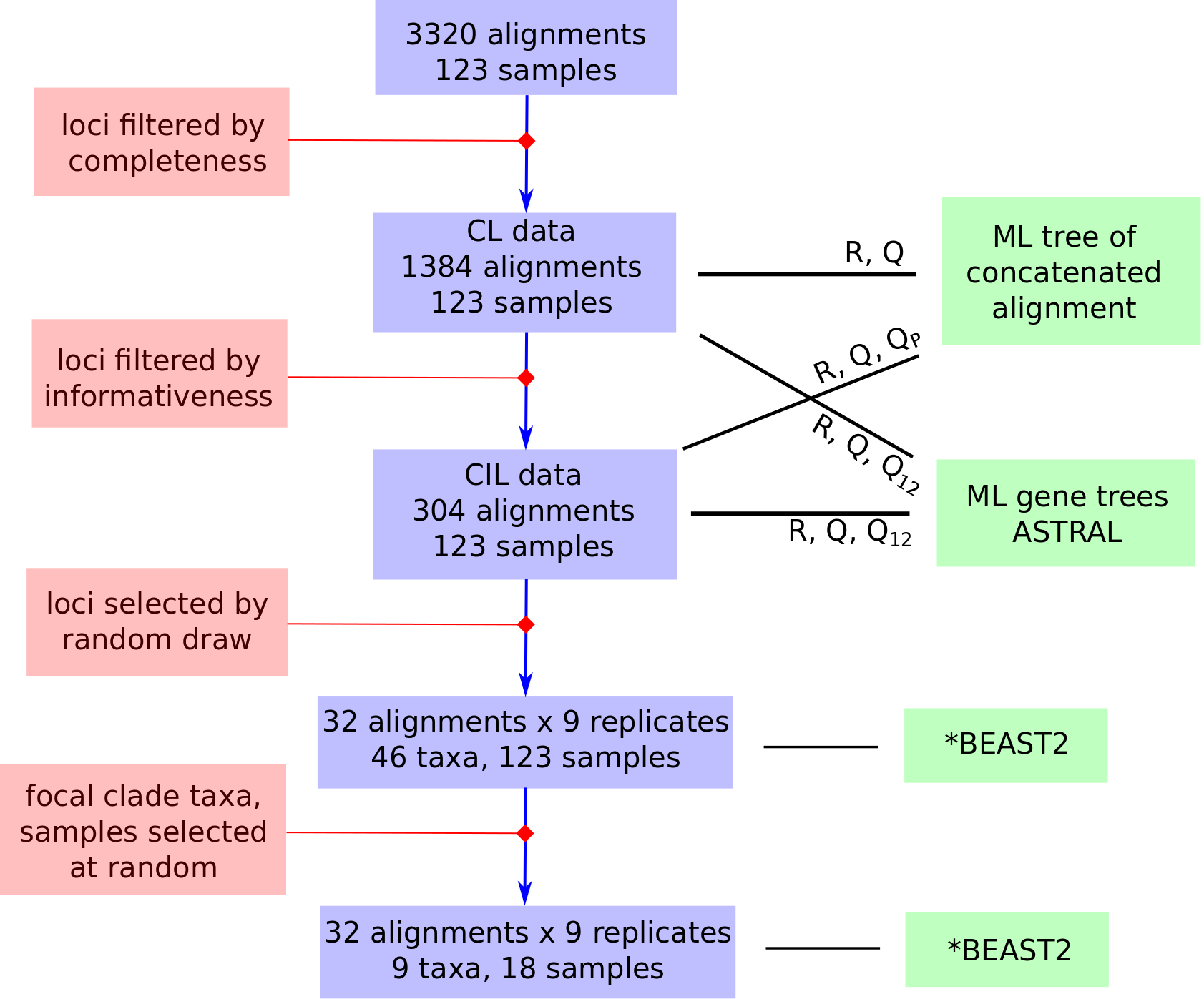


Fig. S1. A guide to the phylogenetic analyses performed in this study (see text for more details of methods). We sequenced a total of 3320 loci. After making alignments, we had 1384 loci with highly complete data, and designate these CL. Of these, we identified a set of 304 loci that were also highly phylogenetically informative, and these are designated CIL. We performed both concatenated and summary coalescent (ASTRAL) analyses of the CL and CIL data. We checked these analyses were robust to different analytical approaches by using different implementations (RAxML, R; IQTREE, Q), partitioning (Q_P_) and excluding third codon positions (Q_12_). We also chose 9 sets of 32 loci at random (without replacement) from the CL data, and for each of these, performed a species tree analysis using StarBEAST2. Finally, we performed an experiment that systematically manipulated the way alleles were selected to represent taxa in StarBEAST2 analyses, and ran these analyses for a focal clade of 9 taxa.


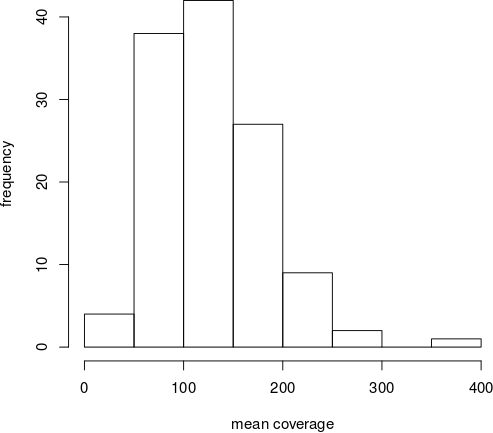


Fig. S2. Histogram showing the distribution of sequencing coverage (averaged across the assembled loci) for 123 skink samples.


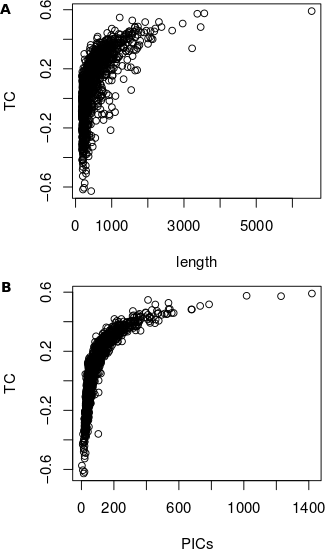


Fig. S3. The relationship between the relative tree certainty score (TC; including all conflicting bipartitions [50]) of the CL alignments, and their (A) length and (B) number of parsimony informative characters (PICs).
